# Assessment of Clinical Features in HIV-Infected Patients with Acute Coronary Syndromes Undergoing Percutaneous Coronary Intervention in China

**DOI:** 10.1155/2022/8351304

**Published:** 2022-06-28

**Authors:** Ying Liu, Yongfu Chen, Yiwei Hao, Jing Xiao, Bei Li, Leidan Zhang, Junyan Han, Hongxin Zhao

**Affiliations:** ^1^Capital Medical University Affiliated Beijing Ditan Hospital, Beijing, China; ^2^Peking University Ditan Teaching Hospital, Beijing, China; ^3^Beijing Key Laboratory of Emerging Infectious Diseases, Institute of Infectious Diseases, Beijing Ditan Hospital, Capital Medical University, Beijing, China

## Abstract

**Objectives:**

We aimed to compare coronary risk factors, burden of coronary artery disease (CAD), and 1-year prognosis of people living with HIV (PLWH) and HIV-negative controls who underwent percutaneous coronary intervention (PCI) for acute coronary syndromes (ACSs).

**Background:**

Cardiovascular disease is drawing more and more attention in PLWH since effective antiretroviral therapy (ART) has been available. Clinical characteristics and outcomes of PLWH undergoing PCI for ACS in China remain unknown.

**Methods:**

We compared demographic characteristics, angiographic features, and 1-year outcomes of 48 PLWH versus 48 HIV-negative controls matched for age (±2 years), sex, diabetes mellitus, and year of PCI (±2 years) in Beijing Ditan Hospital, Capital Medical University from January 2008 to November 2020.

**Results:**

In PLWH (mean age: 53.6 ± 10.6 years, 95.8% male, and 79.2% on ART), high-density lipoprotein cholesterol was lower than in HIV-negative controls; however, the statin use was more common, the incidence of hypertension was lower, and low-density lipoprotein cholesterol, and the body mass index were significantly lower than in controls. Two groups had a similar extent of coronary atherosclerosis as measured by the presence of multivessel diseases and the median Gensini score; however, lesions of PLWH were longer and were more likely to locate at the proximal segment of the coronary artery. In addition, the risk of major adverse cardiac and cerebrovascular events at 1 year was similar in both groups.

**Conclusion:**

PLWH undergoing PCI displayed similar CAD burden and 1-year prognosis compared with HIV-negative patients. Early detection of cardiovascular risk factors and appropriate secondary prevention of CAD in PLWH might alleviate the risk of severe adverse cardiovascular events.

## 1. Introduction

The introduction of antiretroviral therapy (ART) resulting in care of HIV has dramatically changed the natural history of people living with HIV (PLWH) [1, 2]. Today, PLWH have a life expectancy not significantly different from HIV-negative people. However, concerns remain about all the comorbidities associated with aging and the effects of chronic therapies in this population, including coronary artery disease (CAD) [3].

Chronic immune activation, inflammation, metabolic disturbances secondary to antiretroviral drugs, and high prevalence of traditional risk factors are involved in the development of premature atherosclerosis and atherothrombosis in PLWH [4, 5]. However, studies focusing on percutaneous coronary intervention (PCI) in PLWH with acute coronary syndrome (ACS) are sparse [6], and discrepancies have been reported in coronary risk factors, angiographic features, and prognosis during the acute and longer-term phases [7–10]. The aim of our study was to compare coronary risk factors, angiographic features, and 1-year clinical outcomes post PCI of PLWH and HIV-negative patients with ACS.

## 2. Methods

### 2.1. Study Design and Population

The data for all PLWH admitted for ACS in Beijing Ditan Hospital, Capital Medical University from January 2008 to December 2020 were collected from the Tongren medical information system, which included medical record data for all inpatient hospitalizations in Beijing Ditan Hospital since 2008. Diagnoses at discharge are coded according to the International Classification of Diseases, ninth revision (ICD-9). All PLWH diagnosed with unstable angina (UA, *n* = 27) or myocardial infarction (MI, *n* = 50) were included in the study. After excluding those underwent plain old balloon angioplasty alone (*n* = 7), coronary angiography alone (*n* = 16), thrombolysis alone (*n* = 3), and failed PCI (*n* = 3), our study population included 48 PLWH who underwent PCI with at least one stent. As age (±2 years), sex, diabetes mellitus, and year of PCI (±2 years) are strong risk factors for cardiovascular disease, we applied the method of individual matching to enroll the HIV-negative patients who underwent PCI due to MI or UA as the control group by 1 : 1 matching. The study was approved by the Human Science Ethical Committee of Beijing Ditan Hospital, Capital Medical University (No. 2021-021-02).

### 2.2. Data Collection

Baseline characteristics, quantitative CAG, and one-year major adverse cardiac and cerebrovascular events (MACCEs) were compared in PLWH with matched HIV-negative patients. MACCE were defined as recurrent ACS, reintervention, stroke, and all-cause death. Data were also collected concerning HIV infection. Loss of follow-up was defined as no clinic visit every 3 months and no inpatient record within 1 year. We assessed ACS severity using a 6-month mortality prediction scale, and the GRACE score was performed the last week before discharge [11].

#### 2.2.1. Baseline Characteristics

Baseline characteristics included demographic characteristic, medical history, physical examination, the laboratory data, and echocardiographic parameters on admission.

#### 2.2.2. HIV-Related Characteristics

Data on HIV infection were collected, including CD4 counts and HIV load (VL) within 6 months before ACS, prior exposure to ART, time since HIV diagnosis, ART regimen, history of opportunistic infections, and comorbidities.

#### 2.2.3. Angiographic Data Collection

The characteristics and severity of CAD were assessed according to lesion characteristics, the number of diseased vessels, the thrombolysis in myocardial infarction (TIMI) flow grade before and after PCI, and the Gensini score. Lesion characteristics included the lesion length (>20 mm as long lesions), lesion location, and degree of stenosis with or without a thrombus. The diseased vessel was defined as the vessel with stenosis. The culprit vessel was defined as the vessel that was registered as the primary-treated vessel during PCI [12]. For the Gensini score, grading scales were applied according to the degree of stenosis for each coronary lesion. The score was further multiplied depending on the artery type (left anterior descending, left circumflex, and right coronary) and the location (proximal, mid, and distal). The final Gensini score was the summation of each individual lesion score [13].

### 2.3. Statistical Analysis

Continuous variables were presented as the mean ± standard deviation (SD) and the median (interquartile range (IQR)) and were compared using Student's *t*-test and the Wilcoxon rank-sum test, respectively. Categorical variables were presented as frequency (percentage) and compared using the chi-squared test. Survival curves were analyzed using the Kaplan–Meier curves.

Multivariate Cox regression analysis was performed to determine the hazard ratio (HR) for the one-year MACCE. The body mass index (BMI), hypertension, and use of statins on admission were used for our multivariate analysis. *P* values <0.05 were considered statistically significant. All analyses were performed using SPSS statistical software version 26.0.

## 3. Results

### 3.1. Baseline Characteristics

A total of 48 PLWH (95.8% men) with confirmed ACS who underwent PCI were enrolled in the present analysis. The mean age was 53.6 years, with 43.8% of patients having diabetes mellitus. A total of 16 PLWH presented as ST-segment-elevation myocardial infarction (STEMI, 33.3%), 11 PLWH presented as non-ST-segment-elevation myocardial infarction (NSTEMI, 22.9%), and 21 PLWH presented as UA (43.8%). The incidence of UA tended to be higher in PLWH but not statistically significant (43.8% vs. 33.3%, *P*=0.577).  Table 1 depicts the main clinical characteristics of two groups. The proportions of current smokers (56.3% vs. 66.7%) and of those with chronic kidney disease (4.2% vs. 8.3%) were similar to the two groups (all *P* values >0.05). The incidence of hypertension was significantly higher in the HIV-negative group (64.6% vs. 37.5%, *P*=0.008), but the blood pressure at admission was similar (*P*=0.315). A family history of CVD was more common but not significant (41.7% vs. 25%, *P*=0.083) in the HIV-negative group. Prescription of most drugs for secondary prevention of CVD (aspirin, clopidogrel, *β* receptor blockers, and renin angiotensin aldosterone system (RAAS) inhibitors) at admission was comparable between groups (all *P* values >0.05). However, PLWH received statin more frequently (47.9% vs. 10.4%, *P* < 0.001). They had a lower BMI (24.7 ± 4.5 kg/m^2^ vs. 26.8 ± 3.8 kg/m^2^, *P* < 0.001) than HIV-negative controls.

As compared to HIV-negative controls, low-density lipoprotein cholesterol (LDL-C 1.95 ± 0.77 mmol/L vs. 2.69 ± 0.99 mmol/L, *P* < 0.001) and high-density lipoprotein cholesterol (HDL-C 0.79 ± 0.22 mmol/L vs. 0.92 ± 0.21 mmol/L, *P*=0.006) were significantly lower in PLWH. They also showed significantly decreased fasting blood-glucose (7.27 ± 2.76 mmol/L vs. 8.87 ± 3.53 mmol/L, *P*=0.017) but no difference in hemoglobin A1c (HbA1c). Echocardiographic findings on admission did not differ, with the comparable left ventricular ejection fraction (LVEF, 52% ± 14% vs. 56% ± 9%, *P*=0.069), left ventricular mass index (LVMI, 96 g/m^2^ (IQR, 77–114) vs. 88 g/m (IQR, 74–104), *P*=0.137), and incidence of left ventricular diastolic dysfunction (LVDD, 60.4% vs. 71.7%, *P*=0.247) between groups.

### 3.2. HIV-Related Characteristics

Table 2 shows the main parameters regarding HIV infection. A total of 38 (79.2%) PLWH were on ART with the median treatment duration of 4 years (IQR 2–9). Of these patients, non-nucleoside reverse transcriptase inhibitors (NNRTIs) and nucleoside reverse transcriptase inhibitors (NRTIs) were the most common antiretroviral drugs (31.6% and 47.4%, respectively). Only 15.8% of them were taking protease inhibitors (PIs). The median CD4^+^ T cell counts and VL prior to ACS were 365 (182–631) cells/mL and <40 copies/ml (IQR < 40–37633), respectively. A total of 16 PLWH were syphilis positive, 3 PLWH were hepatitis C virus (HCV) positive, and 9 PLWH had a history of opportunistic infections.

### 3.3. Angiographic Characteristics

Angiographic characteristics are shown in Table 3. Overall, the incidence of multivessel diseases (47.9% vs. 42.8%, *P*=0.793) and the median Gensini score (55 ± 37 vs. 52 ± 27, *P*=0.646) was comparable; however, PLWH were more likely to have a longer total length of lesion (52 mm (33–90) vs. 32 mm (22–59), *P*=0.007) and more stents implanted (2.42 ± 1.66 vs. 1.56 ± 1.11, *P*=0.004). As many patients had more than one diseased vessel, 108 diseased vessels for the PLWH group and 109 diseased vessels for the HIV-negative control group were analyzed. The left anterior descending artery (LAD) was the most common diseased vessel and the culprit vessel in both groups (all *P* values >0.05). Nonsignificant differences were detected with regard to coronary thrombi (4.6% vs. 9.2%, *P*=0.498), diameter stenosis (85% (75%–100%) vs. 80% (60%–100%), *P*=0.149), and TIMI flow grade before PCI (*P*=0.776). As compared to the control group, PLWH were found to have more long lesions (59.2% vs. 38.5%, *P*=0.002) and more proximal lesions (50.9% vs. 35.8%, *P*=0.024).

### 3.4. Characteristics at Discharge and 1-Year MACCE

**Figure 1:**
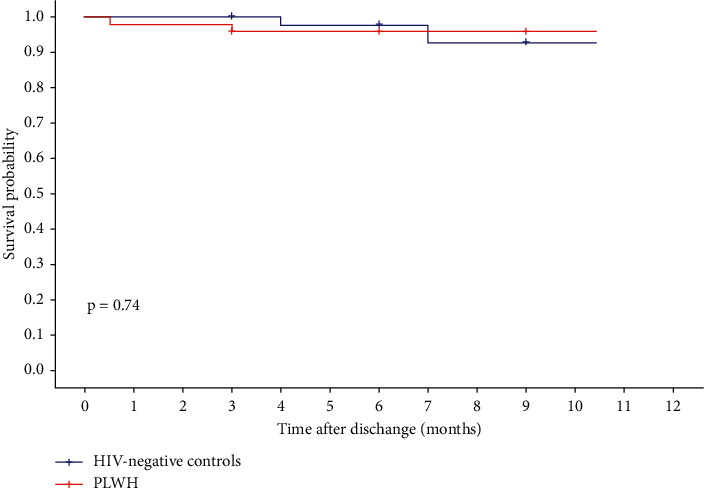
Kaplan–Meier (KM) curves for different HIV status.

The rates of evidence-based medication use on discharge were high in both groups (Table 4), with similar rates of dual antiplatelet therapy (100% vs. 97.9%, *P*=1.000), statin (100% vs. 100%, *P*=1.000), *β* receptor blocker (83.3% vs. 85.4%, *P*=1.000), and RAAS inhibitors (68.8% vs. 79.2%, *P*=1.000). The mean GRACE score at discharge was comparable between the two groups (71.8 ± 24.8 vs. 69.5 ± 19.3, *P*=0.611). Of these patients, 30 PLWH and 37 HIV-negative controls have 1-year follow-up data. The overall incidence (20% vs. 12.5%, *P*=0.688, Table 4) and risk (*P*=0.74, Figure 1) of 1-year MACCE did not differ significantly between the PLWH group and the HIV-negative group. Three PLWH and 3 HIV-negative controls had a recurrent ACS (*P*=1.000). Three PLWH and 1 control required reintervention (*P*=0.318). Two controls experienced stroke. No patient experienced all-cause death. Multivariate Cox regression for major independent factors, including hypertension, BMI, and use of statins on admission, did not alter clinical outcomes (Table 5).

## 4. Discussion

Our study reported the coronary risk factors, angiographic pattern, and clinical outcome of ACS in PLWH undergoing PCI. PLWH were compared with the control group of HIV-negative patients matched for age, sex, diabetes mellitus, and year of PCI. The results suggested PLWH had a similar extent of CAD according to the presence of multivessel disease and the median Gensini score as compared with HIV-negative patients. However, lesions of PLWH were characterised by a longer and more proximal stenosis. The risk of MACCE did not significantly differ between the two groups, even after multivariable adjustments.

HIV infection has become a chronic disease with non-AIDS defining illnesses as the main cause of morbidity and mortality, particularly CAD [14]. PLWH are at increased risk for CAD compared with the general population of the same age [15, 16]. The presence of CAD is usually revealed by the occurrence of ACS, and PCI is the primary method of revascularization in PLWH [6, 17]. Available data suggest the presence of an accelerated process of coronary atherosclerosis in this population due to a higher prevalence of traditional risk factors, chronic inflammation, immune activation, and aging related to HIV infection [18]. In the present study, most of PLWH undergoing PCI were receiving ART. Some of the antiretroviral drugs such as efavirenz and PIs, which are widely used in China, are associated with severe lipodystrophy, disturbed glucose metabolism, and dyslipidemia [19]. However, the prescription rate of the drugs for secondary prevention of CVD in our study was extremely high in PLWH both on admission and at discharge. The incidence of lipid metabolic abnormalities including an elevated serum cholesterol level and hypertriglyceridemia did not show a significant increase in PLWH of our cohort, which might be due to the high rate of statin use. The good adherence of cardiovascular drugs in PLWH of our cohort may benefit from close outpatient follow-up and physicians' awareness of taking intervention to mitigate risk of non-AIDS events. In addition, the prevalence of traditional cardiovascular factors including hypertension and a high BMI was lower in PLWH than that in HIV-negative patients, suggesting the contribution of HIV infection-associated factors in the pathogenesis of CAD. Previous studies have reported that PLWH had a higher prevalence of LVDD and a higher LVMI than those in HIV-negative controls, which might be associated with low nadir and recent CD4 count, higher levels of inflammation, side effects from medication, and the direct effect of virus on the myocardium [20, 21]. However, in patients with ACS, we observed similar echocardiographic findings between groups. The predominant mechanism may be ischemic heart disease and atherosclerosis in the setting of ACS [20].

In the previous studies, the most common presentation is STEMI (29% to 64%), followed by NSTEMI (20% to 48%), and UA (18% to 46%) [4]. The risk of myocardial infarction (MI) increased by 50%–100% in HIV-infected patients compared with control individuals without HIV infection [2, 22, 23], probably due to the increased prevalence of noncalcified coronary atherosclerotic plaque and high-risk morphological plaque [6, 24]. However, whether the type of ACS differs in the HIV-infected population compared with the uninfected population is not obvious. Hsue et al. reported more UA in PLWH, and Boccara et al. described the similar spectrum of ACS in PLWH and HIV-negative patients with a first episode of ACS [7, 25]. In our study, UA was slightly more common in PLWH and STEMI was slightly more common in HIV-negative patients. However, there was no significant difference in these rates between the two groups.

We provided the angiographic description of coronary vasculopathy and lesion characteristics in patients with HIV. The degree of CAD assessed by the number of diseased vessels was reported in PLWH, with 35% to 56% of patients had single-vessel disease, 18% to 28% of patients had double-vessel disease, and 13% to 76% of patients had triple-vessel disease [26–28]. We investigated by quantifying the CAD burden with the presence of multivessel disease as well as the Gensini score and reported similar prevalence of multivessel disease and the median Gensini score in both groups. In addition, the lesions of PLWH were characterised by longer stenosis and were localized in more proximal coronary segments, which were also reported in other studies [8, 29]. The angiographic differences and the discord between the angiographic profile and clinical presentation may reflect substantial differences in the pathogenesis of atherosclerosis in PLWH [8]. In the future, intravascular imaging (intravascular optical coherence tomography, intravascular ultrasound imaging, etc.) would provide more accurate assessments.

The long-term prognosis of PLWH with ACS has been evaluated in a few studies. PLWH exhibited a higher rate of recurrent ischemic events compared with HIV-negative patients, but long-term cardiovascular and total mortality did not differ significantly [7, 26, 30]. In the present study, the risk of overall MACCE at 1 year was similar in both groups. A slightly higher incidence of reintervention was observed in PLWH. The absence of difference in prognosis may be attributed to the high rate of evidence-based medications. This suggests that there is now a need to improve the completeness of secondary prevention medication among PLWH with ACS to ensure that all the potential benefits of secondary prevention are realised.

### 4.1. Study Limitations

The present study had several limitations. First, this study is limited by the small size, single center, and retrospective nature. Second, the rate of loss to follow-up was relatively high, which might have an impact on reliability of results. In addition, a one-year MACCE may not reflect the true picture, and a long-term study may reflect different results.

## 5. Conclusion

In conclusion, our study suggests that PLWH undergoing PCI displayed longer and more proximal lesions but similar CAD burden compared with age and sex matched HIV-negative patients. The 1-year prognosis after PCI does not significantly worsen compared with HIV-negative controls. Taking early detection of cardiovascular risk factors and appropriate secondary prevention of CAD in PLWH might alleviate the risk of severe adverse cardiovascular events.

## Figures and Tables

**Table 1:** The demographic information, previous medical history, and laboratory results at admission compared between PLWH and HIV-negative controls.

	PLWH (*n* = 48)	HIV-negative controls (*n* = 48)	*P* value
Age (years, mean and SD)	53.6 ± 10.6	53.0 ± 10.3	0.777
Male (*n*, %)	46 (95.8%)	46 (95.8%)	1.000
Current smoker (*n*, %)	27 (56.3%)	32 (66.7%)	0.294
BMI (kg/m^2^, mean and SD)	24.7 ± 4.5	26.8 ± 3.8	0.018
Hypertension (*n*, %)	18 (37.5%)	31 (64.6%)	0.008
BP (mean of systolic and diastolic BP (mmHg) ± SD)	102 ± 13	104 ± 13	0.315
Diabetes mellitus (*n*, %)	21 (43.8%)	21 (43.8%)	1.000
On insulin (*n*, %)	6 (12.5%)	5 (10.4%)	0.749
Chronic kidney disease (*n*, %)	2 (4.2%)	4 (8.3%)	0.677
Family history of CVD (*n*, %)	12 (25%)	20 (41.7%)	0.083

*Medications on admission (n, %)*			
Aspirin	14 (29.2%)	9 (18.8%)	0.232
Clopidogrel	10 (20.8%)	5 (10.4%)	0.160
Statin	23 (47.9%)	5(10.4%)	<0.001
Β receptor blocker	8 (16.7%)	3 (6.3%)	0.109
RAAS inhibitor	11 (22.9%)	14 (29.8%)	0.447

*CAD presentation (n, %)*			
STEMI	16 (33.3%)	19 (39.5%)	0.577
NSTEMI	11 (22.9%)	13 (27.0%)	
UA	21 (43.8%)	16 (33.3%)	

*Echocardiographic parameters*			
LVEF (%, mean and SD)	52 ± 14	56 ± 9	0.069
LVDD (*n*, %)	29 (60.4%)	33 (71.7%)	0.247
LVMI (g/m^2^, median and IQR)	96 (77–114)	88 (74–104)	0.137

*Baseline labs (mean and SD/median and IQR)*			
Triglycerides (mmol/L)	1.60 (1.05–2.25)	1.55 (1.10–2.20)	0.781
Total cholesterol (mmol/L)	3.73 ± 1.34	4.37 ± 1.13	0.016
LDL-C (mmol/L)	1.95 ± 0.77	2.69 ± 0.99	<0.001
HDL-C (mmol/L)	0.79 ± 0.22	0.92 ± 0.21	0.006
cTnI (ng/ml	0.21 (0.09–0.92)	0.21 (0.05–2.91)	0.118
BNP (pg/ml)	76 (39–386)	140 (99–236)	0.470
D-dimer (mg/L)	0.28 (0.15–0.69)	0.57 (0.43–0.93)	0.089
Fasting blood-glucose (mmol/L)	7.27 ± 2.76	8.87 ± 3.53	0.017
HbA1c (%)	6.1 (5.5–6.7)	5.9 (5.4–8.2)	0.170
Creatinine (mmol/L)	79 ± 30	78 ± 24	0.871
Leukocyte counts (109/L)	5.64 ± 1.83	9.39 ± 3.61	<0.001
Hemoglobin (g/dL)	13.9 ± 5	14.5 ± 1.4	0.493
CRP (mg/dL)	2.5 (1.2–7.2)	5 (1.2–9.7)	0.722

PLWH: people living with HIV; BMI: body mass index; BP: blood pressure; CVD: cardiovascular disease; SD: standard deviation; IQR: interquartile range; RAAS: renin angiotensin aldosterone system; CAD: coronary artery disease; STEMI: ST-segment-elevation myocardial infarction; NSTEMI: non-ST-segment-elevation myocardial infarction; UA: unstable angina; LVEF: left ventricular ejection fraction; LVDD: left ventricular diastolic dysfunction; LVMI: left ventricular mass index; LDL-C: low-density lipoprotein cholesterol; HDL-C: high-density lipoprotein cholesterol; BNP: brain natriuretic peptide; HbA1c: hemoglobin A1c; CRP: C-reactive protein.

**Table 2:** HIV-related characteristics at admission.

Variables	Statistic value
Receiving ART (*n*, %)	38 (79.2%)
^*∗*^time since ART initiation (years, median, and IQR)	4 (2–9)
History of opportunistic infections (*n*, %)	9 (18.8%)
Syphilis (*n*, %)	16 (33.3%)
HCV (*n*, %)	3 (6.3%)
^*∗∗*^CD4 cell counts before ACS (cells/*μ*l, median, and IQR)	365 (182–631)
^*∗∗∗*^VL before ACS (copies/ml, median, and IQR)	<40 (<40–37633)

^a^ART drugs (*n*, %)	
NRTIs	18 (47.4%)
NNRTIs	12 (31.6%)
PIs	6 (15.8%)
Unknown	2 (5.3%)

The symbol ^*∗*^indicates that 38 patients were included receiving ART; the symbol ^*∗∗*^ indicates that the CD4 cell count was only available in 31 patients; the symbol ^*∗∗∗*^ indicates thatVL was only available in 26 patients. ART: antiretroviral therapy; IQR: interquartile range; HCV: hepatitis C virus; ACS: acute coronary syndrome; VL: HIV load; NNRTIs: non-nucleoside reverse transcriptase inhibitors; NRTIs: nucleoside reverse transcriptase inhibitors; PIs: protease inhibitors.

**Table 3:** Angiographic characteristics in PLWH and HIV-negative controls.

	PLWH (*n* = 48)	HIV-negative controls (*n* = 48)	*P* value
Number of diseased vessels (*n*, %)			0.793
1-vessel	13 (27.1%)	12 (25.0%)	
2-vessel	12 (25.0%)	15 (31.3%)	
3-vessel	23 (47.9%)	21 (42.8%)	
Gensini score (mean and SD)	55 ± 37	52 ± 27	0.646
Total length of lesion (mm, median, and IQR)	52 (33–90)	32 (22–59)	0.007
Total number of stents (mean and SD)	2.42 ± 1.66	1.56 ± 1.11	0.004

	Diseased vessels of PLWH (*n* = 108)	Diseased vessels of HIV-negative controls (*n* = 109)	*P* value

Culprit lesion location (*n*, %)			0.194
Left main	0 (0%)	0 (0%)	
LAD	33 (49.3%)	32 (58.2%)	
LCX	16 (23.9%)	10 (18.2%)	
RCA	18 (26.9%)	13 (23.6%)	
Lesion location (*n*, %)			0.401
Left main	1 (0.9%)	1 (0.9%)	
LAD	39 (36.1%)	44 (40.4%)	
LCX	32 (29.6%)	30 (27.5%)	
RCA	36 (33.3%)	34 (31.2%)	
Long lesion (*n*, %)	64 (59.2%)	42 (38.5%)	0.002
Location within artery (*n*, %)			
Proximal	55 (50.9%)	39 (35.8%)	0.024
Mid	34 (31.5%)	46 (42.2%)	0.102
Distal	19 (17.6)	24 (22.0%)	0.413
Thrombus (*n*, %)	5 (4.6%)	10 (9.2%)	0.498
Diameter stenosis (%, median and IQR)	85 (75–100)	80 (60–100)	0.149
TIMI flow before PCI (*n*, %)			0.776
0	25 (23.1%)	25 (22.9%)	
1	3 (2.8%)	5 (4.6%)	
2	0 (0%)	0 (0%)	
3	80 (71.3%)	79 (69.7%)	
TIMI flow after PCI (*n*, %)			1.000
0	0 (0%)	0 (0%)	
1	0 (0%)	0 (0%)	
2	0 (0%)	0 (0%)	
3	108 (100%)	109 (100%)	

PLWH: people living with HIV; SD: standard deviation; IQR: interquartile range; LAD: left anterior descending artery; LCX: left circumflex coronary artery; RCA: right coronary artery; TIMI: thrombolysis in myocardial infarction; PCI: percutaneous coronary intervention.

**Table 4:** GRACE score and medications at discharge and the one-year MACCE incidence.

	PLWH (*n* = 48)	HIV-negative controls (*n* = 48)	*P* value
GRACE score at discharge (mean and SD)	71.8 ± 24.8	69.5 ± 19.3	0.611

*Medications at discharge (n, %)*			
DAPT	48 (100%)	47 (97.9%)	1.000
Statin	48 (100%)	48 (100%)	1.000
*β* receptor blocker	40 (83.3%)	41 (85.4%)	1.000
RAAS inhibitor	33 (68.8%)	38 (79.2%)	0.245

	PLWH (*n* = 30)	HIV-negative controls (*n* = 37)	*P* value

One-year MACCE (*n*, %)	6 (20%)	6 (12.5%)	0.688
Recurrent ACS (*n*, %)	3 (10%)	3 (8.1%)	1.000
Reintervention (*n*, %)	3 (10%)	1 (2.7%)	0.318
Stroke (*n*, %)	0 (0%)	2 (1.1%)	0.498
All-cause death (*n*, %)	0 (0%)	0 (0%)	1.000

MACCEs: major adverse cardiac and cerebrovascular events; PLWH: people living with HIV; DAPT: dual antiplatelet therapy; RAAS: renin angiotensin aldosterone system; ACS: acute coronary syndrome.

**Table 5:** Multivariate logistic regression analysis of 1-year MACCE.

	HR	95% CI	*P* value
HIV status	0.65	0.17–2.50	0.53
BMI	0.98	0.85–1.14	0.82
Hypertension	0.42	0.17–1.64	0.21
Use of statin on admission	1.79	0.45–7.12	0.41

MACCEs: major adverse cardiac and cerebrovascular events; HR: hazard ratio; CI: confidence interval; BMI: body mass index.

## Data Availability

The data that support the findings of this study are available from the corresponding author upon reasonable request.

## References

[1] Trickey A., May M. T., Vehreschild J. (2017). Survival of HIV-positive patients starting antiretroviral therapy between 1996 and 2013: a collaborative analysis of cohort studies. *The Lancet HIV*.

[2] Thienemann F., Sliwa K., Rockstroh J. K. (2013). HIV and the heart: the impact of antiretroviral therapy: a global perspective. *European Heart Journal*.

[3] Marcus J. L., Leyden W. A., Alexeeff S. E. (2020). Comparison of overall and comorbidity-free life expectancy between insured adults with and without HIV infection. *JAMA Network Open*.

[4] Boccara F., Meuleman S. L. C., Ederhy S. (2013). HIV and coronary heart disease. *Journal of the American College of Cardiology*.

[5] Stein J. H., Hsue P. Y. (2012). Inflammation, immune activation, and CVD risk in individuals with HIV infection. *JAMA*.

[6] Bernelli C., Anaziand G. B. D., Cerrato E. (2020). Cardiovascular events recurrence and coronary artery disease in HIV patients: the price we have to pay for the chronicization of the disease. *Canadian Journal of Cardiology*.

[7] Boccara F., Mary-Krause M., Teiger E. (2011). Acute coronary syndrome in human immunodeficiency virus-infected patients: characteristics and 1 year prognosis. *European Heart Journal*.

[8] Theodoropoulos K., Ennuniand M. M., Sartori S. (2017). Quantitative angiographic characterisation of coronary artery disease in patients with human immunodeficiency virus (HIV) infection undergoing percutaneous coronary intervention. *Eurointervention*.

[9] Segev A., Cantor W. J., Strauss B. H. (2006). Outcome of percutaneous coronary intervention in HIV-infected patients. *Catheterization and Cardiovascular Interventions*.

[10] Lorgis L., Ottenetand J. C., Molins G. (2013). Outcomes after acute myocardial infarction in HIV-infected patients. *Circulation*.

[11] Eagle K. A., Lim M. J., Dabbous O. H. (2004). A validated prediction model for all forms of acute coronary syndrome. *JAMA*.

[12] Karami M., Etersand E. P., Lagrand W. (2021). Outcome and predictors for mortality in patients with cardiogenic shock: a Dutch nationwide registry-based study of 75,407 patients with acute coronary syndrome treated by PCI. *Journal of Clinical Medicine*.

[13] Gensini G. G. (1983). A more meaningful scoring system for determining the severity of coronary heart disease. *The American Journal of Cardiology*.

[14] Ingle S. M., Ayand M. T. M., Gill M. J. (2014). Impact of risk factors for specific causes of death in the first and subsequent years of antiretroviral therapy among HIV-infected patients. *Clinical Infectious Diseases*.

[15] Kaplan R. C., Kingsley L. A., Sharrett A. R. (2007). Ten-year predicted coronary heart disease risk in HIV-infected men and women. *Clinical Infectious Diseases*.

[16] Saves M., Chene G., Ducimetiere P. (2003). Risk factors for coronary heart disease in patients treated for human immunodeficiency virus infection compared with the general population. *Clinical Infectious Diseases*.

[17] Pearce D., Ani C. A. Y., Espinosa-Silva Y. (2012). Comparison of in-hospital mortality from acute myocardial infarction in HIV sero-positive versus sero-negative individuals. *The American Journal of Cardiology*.

[18] Reinsch N., Euhausand K. N., Esser S. (2012). Are HIV patients undertreated? cardiovascular risk factors in HIV: results of the HIV-HEART study. *European Journal of Preventive Cardiology*.

[19] Lagathu C., Béréziat V., Gorwood J. (2019). Metabolic complications affecting adipose tissue, lipid and glucose metabolism associated with HIV antiretroviral treatment. *Expert Opinion on Drug Safety*.

[20] Hsue P. Y., Hunt P. W., Ho J. E. (2010). Impact of HIV infection on diastolic function and left ventricular mass. *Circulation: Heart Failure*.

[21] Okeke N. L., Alenezi F., Bloomfield G. S. (2018). Determinants of left ventricular hypertrophy and diastolic dysfunction in an HIV clinical cohort. *Journal of Cardiac Failure*.

[22] Triant V. A., Lee H., Hadigan C., Grinspoon S. K. (2007). Increased acute myocardial infarction rates and cardiovascular risk factors among patients with human immunodeficiency virus disease. *Journal of Clinical Endocrinology and Metabolism*.

[23] Freiberg M. S., Chang C. C., Kuller L. H. (2013). HIV infection and the risk of acute myocardial infarction. *JAMA Internal Medicine*.

[24] Zanni M. V., Abbara S., Lo J. (2013). Increased coronary atherosclerotic plaque vulnerability by coronary computed tomography angiography in HIV-infected men. *AIDS*.

[25] Hsue P. Y., Erickson K. G. S., MacGregor J. S., Younes N., Shergill A., Waters D. D. (2004). Clinical features of acute coronary syndromes in patients with human immunodeficiency virus infection. *Circulation*.

[26] Matetzky S., Kar M. D. S., Noc M. (2003). Acute myocardial infarction in human immunodeficiency virus-infected patients. *Archives of Internal Medicine*.

[27] Boccara F., Cohen E. T. A., Ederhy S. (2006). Percutaneous coronary intervention in HIV infected patients: immediate results and long term prognosis. *Heart*.

[28] Ren X., Kwan M. T. D. M., Nguyen K., Shaw R. E., Hui P. Y. (2009). Comparison of outcomes using bare metal versus drug-eluting stents in coronary artery disease patients with and without human immunodeficiency virus infection. *The American Journal of Cardiology*.

[29] Escaut L., Monsuez J. J., Chironi G. (2003). Coronary artery disease in HIV infected patients. *Intensive Care Medicine*.

[30] D’Ascenzo F., Cerrato E. C. G., Biondi-Zoccai G. (2012). Acute coronary syndromes in human immunodeficiency virus patients: a meta-analysis investigating adverse event rates and the role of antiretroviral therapy. *European Heart Journal*.

